# Anticoagulation with Nafamostat Mesilate During Impella Support: A Case Report

**DOI:** 10.3390/medicina61020309

**Published:** 2025-02-10

**Authors:** Makiko Nakamura, Teruhiko Imamura, Yuki Hida, Toshihide Izumida, Masaki Nakagaito, Saori Nagura, Toshio Doi, Koichiro Kinugawa

**Affiliations:** 1 The Second Department of Internal Medicine, University of Toyama, 2630 Sugitani, Toyama 930-0194, Japan; nakamura@med.u-toyama.ac.jp (M.N.); time.to.control5@gmail.com (Y.H.); mgaito128@gmail.com (M.N.); kinugawa@med.u-toyama.ac.jp (K.K.); 2 Department of Cardiovascular Surgery, University of Toyama, 2630 Sugitani, Toyama 930-0194, Japan; saori79@med.u-toyama.ac.jp (S.N.); tdoi@med.u-toyama.ac.jp (T.D.)

**Keywords:** thrombocytopenia, ECMO, ENaC, hyponatremia, case report

## Abstract

Achieving an optimal balance between thrombosis prevention and bleeding risk during temporary mechanical circulatory support remains a significant clinical challenge. Effective anticoagulation management that ensures device functionality while minimizing major bleeding events should have the potential to improve short-term outcomes. Here, we report the successful use of nafamostat mesilate (NM) as an anticoagulant during Impella support in two male patients with advanced heart failure and cardiogenic shock. NM therapy resulted in improved thrombocytopenia without the occurrence of major bleeding or thrombotic events. However, NM-related hyponatremia was observed, highlighting the need for careful monitoring during its administration and further cumulative evidence to validate optimal NM therapy during temporary mechanical circulatory supports.

## 1. Introduction

The utilization of mechanical circulatory support (MCS) has significantly increased in patients with advanced heart failure and cardiogenic shock over the past two decades [1,2,3]. In the DANGER Shock Trial, Impella support combined with standard care demonstrated superior six-month survival rates compared to standard care alone in patients with acute myocardial infarction-related cardiogenic shock [4]. However, the incidence of moderate to severe bleeding was notably higher in the Impella plus standard care group [4]. In addition to major bleeding, complications such as hemolysis, infection, and thrombocytopenia remain unresolved challenges associated with temporary MCS therapies [5,6,7]. Thrombocytopenia, a prevalent yet poorly understood complication during temporary MCS therapy, increases the risk of bleeding and necessitates platelet transfusion [6,8]. The severity of thrombocytopenia has been reported to vary depending on the type of MCS device used, including veno-arterial extracorporeal membrane oxygenation (ECMO), veno-venous ECMO, Impella 5.5, and intra-aortic balloon pump (IABP). Importantly, thrombocytopenia is generally reversible following the explantation of MCS devices [6].

Heparin is the standard anticoagulant for managing temporary MCS, but alternative agents are required in certain cases [9]. Heparin-induced thrombocytopenia (HIT), thrombosis, bleeding, and heparin resistance present diagnostic and therapeutic challenges in patients undergoing MCS therapy [9]. In such situations, a bicarbonate-based purge solution may serve as an alternative to a heparinized purge solution during Impella support [10].

Nafamostat mesilate (NM), a synthetic low-molecular-weight serine protease inhibitor, has been employed as an anticoagulant in patients requiring ECMO support or hemodialysis, particularly those with bleeding risks or heparin-related adverse effects [11,12]. NM possesses a short half-life (5–8 min) and inhibits coagulation and platelet aggregation by inactivating plasmin, thrombin, and activated coagulation factors XIIa and Xa, and by interfering with fibrinolysis, complement components C1r and C1s, trypsin, and kallikrein [9,11]. Additionally, NM suppresses factor Xa generation mediated by the interaction between tissue factor and factor V in a concentration-dependent manner, acting as a regional anticoagulant within ECMO circuits. However, NM and its metabolites also inhibit Na–K ATPase-dependent pathways, impairing potassium secretion and potentially causing hyperkalemia [11]. Consequently, optimal anticoagulation strategies using NM during MCS therapy remain inadequately defined.

Herein, we describe two cases in which patients undergoing Impella support were treated with NM. Both patients experienced improvements in thrombocytopenia after initiating NM, without major bleeding or thrombotic events.

## 2. Case Report

### 2.1. Case 1 (Advanced Heart Failure with Ischemic Cardiomyopathy)

#### 2.1.1. On Admission

A 70-year-old male patient with ischemic cardiomyopathy presented with acute decompensated heart failure. At the referring hospital, his left ventricular ejection fraction was measured at 20%, and coronary angiography revealed three-vessel disease with a chronic total occlusion of the right coronary artery. Due to hemodynamic instability, intravenous dobutamine (4.5 μg/kg/min) and noradrenaline (0.03 μg/kg/min) were initiated alongside mechanical ventilation and IABP support. The patient was subsequently referred to our institute for advanced and intensive management.

#### 2.1.2. Impella 5.5 Initiation with NM Administration

The patient’s body surface area and body mass index were 1.89 m^2^ and 28.7 kg/m^2^, respectively. A chest X-ray revealed pulmonary congestion, and right heart catheterization demonstrated an elevated pulmonary arterial wedge pressure (26 mmHg) with a pulmonary artery pulsatility index of 1.23. The IABP was upgraded to Impella 5.5, allowing for the discontinuation of intravenous noradrenaline.

**Figure 1:**
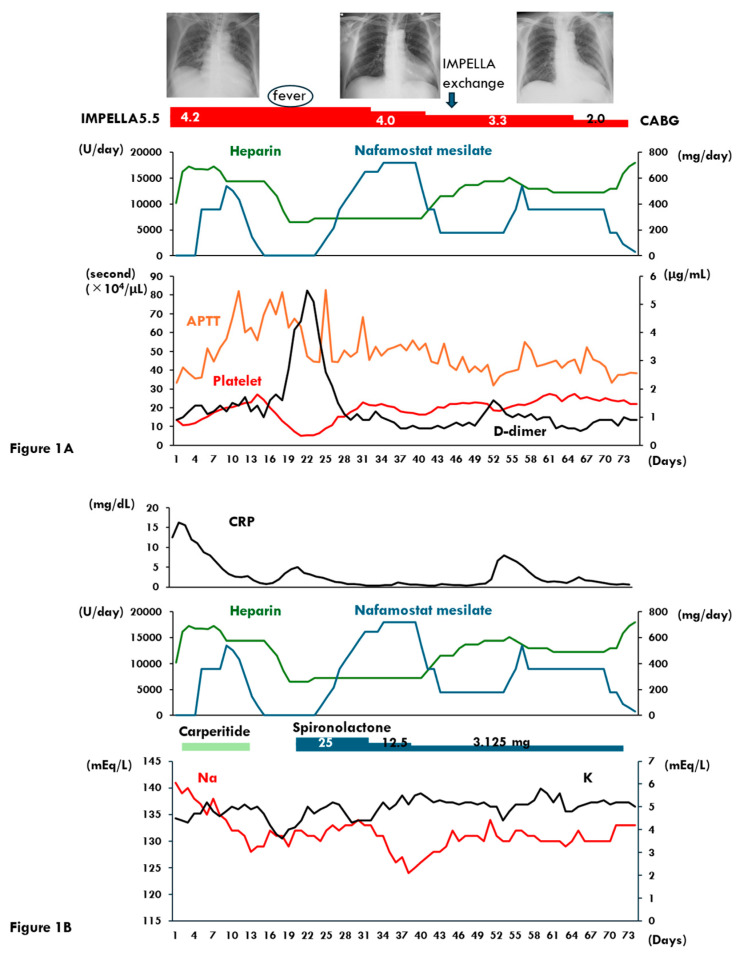
Clinical course after initiation of Impella 5.5 support until coronary artery bypass grafting in Case 1 is shown. (**A**) Transition of the chest X-ray, administered dose of heparin and nafamostat mesilate, the levels of activated partial thrombin time, d-dimer, and platelet count are shown; (**B**) transition of the levels of c-reactive protein, serum sodium, and serum potassium are shown. APTT, activated partial thrombin time; CRP, c-reactive protein; Na, sodium; K, potassium.

On day 3, intravenous carperitide, which is a recombinant atrial natriuretic peptide and has a class IIa recommendation in the current Japanese Circulation Society guidelines [13], was initiated and the patient’s lung congestion gradually improved. However, his platelet count declined to 11 × 10^4^/μL, despite no prolongation in activated partial thromboplastin time (APTT), which remained at 36 s, despite increasing doses of heparin. Consequently, NM was introduced on day 4. Following this transition, both the platelet count and APTT levels improved (Figure 1A). Mechanical ventilation was successfully weaned off by day 12. However, the serum potassium level increased to 5.1 mEq/L on the same day, prompting the discontinuation of NM on day 14 (Figure 1B).

#### 2.1.3. Hyponatremia and NM Dose Adjustment

On day 6, the patient’s urine sodium and potassium concentrations were 147 mEq/L and 65 mEq/L, respectively. By day 13, his serum sodium level had decreased to 129 mEq/L. Intravenous carperitide was discontinued on day 15, considering the pharmacological effect of natriuresis (Figure 1B).

On day 19, the patient developed a fever accompanied by thrombocytopenia (5.1 × 10^4^/μL) and elevated d-dimer levels. Antibiotic therapy was initiated, and NM (360–720 mg/day) was reintroduced on day 24 (Figure 1A). The fever resolved, d-dimer levels decreased, and platelet counts improved. However, serum sodium levels continued to decline, reaching 124 mEq/L on day 38, while serum potassium levels fluctuated around 5 mEq/L. In response, spironolactone was reduced from 25 mg to 3.125 mg daily on day 40 (Figure 1B).

Given the potential association between hyponatremia and NM administration, the NM dose was reduced from 720 mg/day to 180 mg/day on day 43. However, APTT levels gradually declined, necessitating an increase in the NM dose to 360 mg/day on day 55. To stabilize serum sodium levels, saline infusion therapy was initiated on day 61.

From day 70 onward, the NM dose was tapered to 60–180 mg/day. Subsequently, the patient’s serum sodium levels stabilized around 130 mEq/L. By day 72, his urine sodium level had increased to 174 mEq/L, while urine potassium levels decreased to 16 mEq/L.

Impella exchange and revascularization:

The patient’s hemodynamic stability was maintained under Impella 5.5 support. An attempt at percutaneous coronary intervention to the proximal left anterior descending artery was performed but was unsuccessful. Due to an exit-site infection associated with the Impella 5.5 device, the device was replaced on day 45.

Subsequently, the patient underwent coronary artery bypass grafting on day 75 while remaining on Impella 5.5 support. The Impella 5.5 device was successfully removed on day 91. His post-operative course was uneventful, and he was transferred back to the referring hospital. The patient was later discharged home in stable condition.

### 2.2. Case 2 (Fulminant Myocarditis with Thrombocytopenia)

#### 2.2.1. On Admission

A 62-year-old male patient with fulminant myocarditis was referred to our hospital. His body surface area and body mass index were 1.73 m^2^ and 20.7 kg/m^2^, respectively. He initially presented at the referring hospital with a complete atrioventricular block and reduced left ventricular ejection fraction of 30%, requiring temporary pacing and IABP support.

Upon arrival at our facility, his systolic blood pressure was 89 mmHg, and a chest X-ray revealed severe pulmonary congestion. Laboratory evaluations indicated significant end-organ dysfunction, with lactate levels of 7.8 mmol/L, serum creatinine of 2.7 mg/dL, total bilirubin of 1.9 mg/dL, a prothrombin time international normalized ratio of 2.5, severe thrombocytopenia (1.9 × 10^4^/μL), and elevated d-dimer levels (19.0 μg/mL).

**Figure 2:**
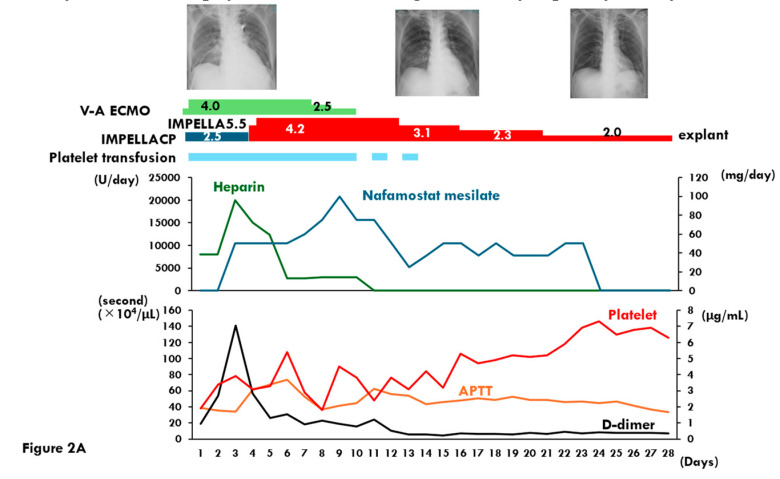

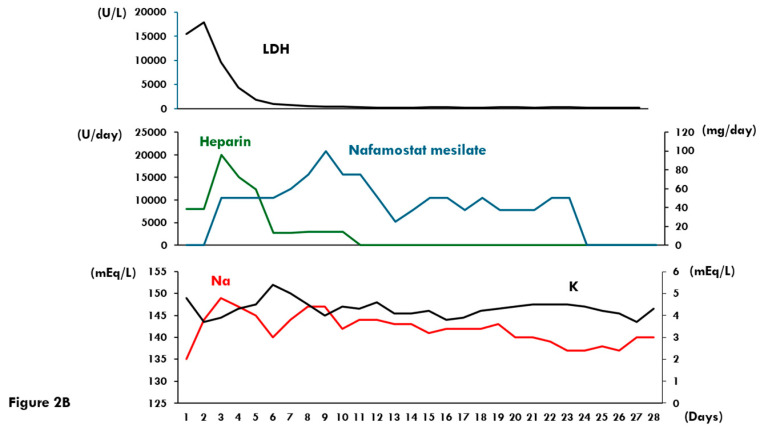
Clinical course after initiation of Impella and veno-arterial extracorporeal membrane oxygenation support until removal in Case 2 is shown. (**A**) transition of the chest X-ray, administered dose of heparin and nafamostat mesilate, the levels of activated partial thrombin time, d-dimer, and platelet count are shown; (**B**) transition of the levels of lactate dehydrogenase, serum sodium, and serum potassium are shown. LDH, lactate dehydrogenase; Na, sodium; K, potassium.

Mechanical ventilation, veno-arterial ECMO, and Impella CP support were initiated following the removal of the IABP (Figure 2A). The patient was also diagnosed with bacteremia caused by *Klebsiella aerogenes* upon admission, and appropriate antibiotic therapy commenced. Testing for anti-HIT antibodies was negative, and an endomyocardial biopsy confirmed a diagnosis of lymphocytic myocarditis.

#### 2.2.2. NM Initiation, Impella Upgrade, and ECMO Explant

Following the initiation of veno-arterial ECMO and Impella CP support, the patient’s lactate levels gradually decreased. However, d-dimer levels increased significantly by day 3, prompting an exchange of the veno-arterial ECMO circuit and the initiation of intravenous NM therapy at 50 mg/day. This intervention resulted in reductions in both d-dimer levels and serum lactate dehydrogenase levels (Figure 2B).

On day 5, the Impella CP device was upgraded to Impella 5.5, and the patient achieved sinus rhythm conversion by day 7. Veno-arterial ECMO was successfully explanted on day 11, marking significant progress in the patient’s recovery.

#### 2.2.3. Low-Dose NM Management

In response to refractory thrombocytopenia, the Impella heparin purge solution was replaced with a bicarbonate-based solution, and systemic anticoagulation was maintained with low-dose NM at 40–80 mg/day. Mechanical ventilation was successfully discontinued on day 14. From day 16 onward, the patient’s platelet count stabilized above 4 × 10^4^/μL, allowing platelet transfusions to be discontinued (Figure 2A).

Throughout this period, the patient’s serum potassium levels remained within the normal range. However, his serum sodium levels decreased slightly to 137 mEq/L, with corresponding urine sodium levels of 66 mEq/L by day 23, leading to the discontinuation of NM therapy (Figure 2B).

The patient’s cardiac function showed progressive recovery, and the Impella 5.5 device was successfully explanted on day 28. He was subsequently transferred back to the referring hospital and later discharged home in stable condition.

## 3. Discussion

### 3.1. Anticoagulation During MCS Therapy

Anticoagulation is essential during ECMO support due to the interaction between the patient’s blood and non-biological artificial surfaces (primarily the oxygenator) and the mechanical forces (high shear stress, stasis, and heat generation) that activate coagulation and thrombin production, leading to an increased risk of bleeding and thrombosis. The use of unfractionated heparin remains a cornerstone of anticoagulation in ECMO, owing to its well-established efficacy, low cost, and the availability of a reversal agent [11]. Various alternatives to unfractionated heparin have also been explored, with NM showing promise in ECMO patients [9,11,14].

In contrast, anticoagulation strategies during Impella support are more complex and vary widely across centers, which may heighten the risk of complications [10]. The use of a heparinized, dextrose-based purge solution is critical for ensuring optimal device function. Although the manufacturer does not officially mandate monitoring of APTT, this assay is widely employed in clinical practice, with most centers targeting therapeutic anticoagulation based on local APTT guidelines.

Bicarbonate-based purge solutions have emerged as a potential alternative, particularly in patients with a heightened risk of bleeding or heparin-related complications. However, systemic anticoagulation with intravenous heparin is often required in these cases. Despite its widespread use, there is a lack of robust evidence to guide the optimal monitoring assay or target range for anticoagulation during Impella support [10].

Future research should prioritize identifying the most effective monitoring strategies and target ranges for intravenous anticoagulation in patients using Impella devices. Such studies would aim to minimize bleeding complications, prevent thrombus formation, and ensure adequate purge pressures, thereby enhancing patient safety and clinical outcomes [10].

### 3.2. Effects of NM on ECMO

NM was developed in Tokyo, Japan by Nivoju Pharmaceutical Co., Ltd., in 1986 [9]. It inhibits various components of the coagulation cascade, including trypsin, thrombin, plasmin, kallikrein, cholesterol enzyme, and coagulation factors VIIa, Xa, and XIIa, as well as several serine proteases within the complement system [9]. NM has been used for anticoagulation in patients with continuous renal replacement therapy in Japan and Korea [14]. In specific Asian countries, NM is approved for indications such as the management of acute symptoms of pancreatitis, anticoagulation in disseminated intravascular coagulation, and anticoagulation during extracorporeal circulation in patients at risk of or experiencing active bleeding.

During the COVID-19 pandemic, NM gained attention for its potential to inhibit transmembrane protease serine 2, a key enzyme facilitating viral entry of the SARS-CoV-2 virus [11,15]. It has been also suggested that NM has anti-inflammatory, antioxidant, and neuroprotective effects [16] that could be variable in severely ill patients [11]. Although NM lacks a specific antidote, its very short half-life (5–8 min) offers an advantage in patients on ECMO, where the balance between thrombotic and bleeding risks can fluctuate rapidly. This characteristic provides a potential benefit over anticoagulants such as argatroban, which has a longer half-life of approximately 45 min [9].

The safety and efficacy of NM as a regional anticoagulation during veno-arterial ECMO is also reported to sustain a lower APTT value compared to that measured at the ECMO site from Korea [14]. In our patient (Case 2), the systemic NM administration during ECMO plus Impella support resulted in an improvement in the thrombocytopenia and a decrease in the d-dimer levels, while APTT levels were prolonged and the dose of concomitant heparin was reduced. It is also reported that veno-arterial ECMO, veno-venous ECMO, and IABP can all induce a significant drop in platelet count, and the degree of platelet count drop is higher in veno-arterial and veno-venous ECMO, leading to an increased risk of thrombocytopenia compared to IABP [6].

NM may have inhibited platelet aggregation and coagulation cascade during ECMO support. Although the best anti-coagulation levels or NM dose remain unknown, there were no major bleeding events nor thrombotic events during ECMO plus Impella support under a relatively lower APTT range (40–70 s) in Case 2.

### 3.3. Anticoagulation with NM During Impella Support

Currently, there are no established data regarding the safety and efficacy of NM therapy during Impella support. The expert committee in the United States has suggested that a sodium bicarbonate-based purge solution (25 mEq per 1000 mL of 5% dextrose) may be preferable in situations where heparin-based purge solutions are contraindicated, such as in cases of HIT or major bleeding [10]. While the discontinuation of heparin in favor of direct thrombin inhibitors like argatroban or bivalirudin has not been validated for Impella use, such approaches may increase both bleeding risk and treatment costs [8].

In Case 1, systemic NM administration in combination with a heparin purge solution led to improvements in thrombocytopenia and facilitated a reduction in heparin dosage. In Case 2, the concomitant use of systemic NM with a bicarbonate-based purge solution following veno-arterial ECMO removal also resulted in the resolution of thrombocytopenia. Notably, in Case 2, APTT levels remained only slightly above the normal range, and no thrombotic events were observed.

The previous study reported that nearly all patients developed thrombocytopenia within 7 days of Impella insertion, with 81% having a >50% decrease, and platelet recovery occurring following device removal [17], clearly demonstrating that it was device-related [8]. Of note, a subgroup of 38% of patients were tested for HIT by enzyme-linked immunosorbent assays, with 8% being positive, but none of these had positive confirmatory results in the confirmatory serotonin release assays [17]. The causes of thrombocytopenia with Impella are multiple, including platelet activation by contact with the nonendothelial surfaces and directly by the high shear forces in the micro-axial pump. High shear forces unfold the von Willebrand factor, exposing platelet-binding sites that may cause further activation of platelets and the formation of platelet thrombi [8].

The preliminary effect of NM for suppressing the platelet-activating factor level elevated by the left ventricular assist device in a canine heart failure model was previously reported [18]. NM may be effective for Impella-related thrombocytopenia, and further investigation is warranted to ensure this hypothesis.

### 3.4. NM-Related Hyponatremia and Hyperkalemia

The reported side effect associated with the use of NM is hyperkalemia, and the incidence was reported as only 15–18% in patients anticoagulated with NM during ECMO support [9,11]. Aguranulocytosis is also reported as a possible side effect of NM therapy in patients receiving continuous renal replacement therapy [19]. The dose of NM used in ECMO patients reported by the studies varied greatly: the average dose ranged from 0.46 to 0.67 mg/kg/h, which are higher than ours (Case 1, 0.09–0.35 mg/kg/h; Case 2, 0.03–0.06 mg/kg/h). On the other hand, in another report, the incidence of hyperkalemia was as high as 47% among patients who received favipiravir and dexamethasone with NM for the treatment of COVID-19, and the duration of NM administration was a significant predictor of hyperkalemia (odds ratio: 1.55, 95% confidence interval: 1.04–2.31) [20]. Not only the concentration or daily dose of NM but also the administered duration of NM may have impact on the induced electrocyte imbalance, including hyperkalemia.

It has been reported that NM and its metabolites inhibit the activity of epidermal sodium channel (ENaC) in renal collecting ducts, inhibit Na+-K+ ATPase in renal collecting ducts, and inhibit aldosterone secretion, resulting in decreased urinary potassium excretion and casing hyperkalemia [20,21]. NM-induced inhibition of ENaC activity could account for the side effects of hyponatremia as well as hyperkalemia [22], like triamterene.

NM is a synthetic low-molecular-weight serine protease inhibitor with a very short half-life (5–8 min), metabolized by the liver and in the bloodstream, and is excreted through the kidneys and bowels [11]. The side effects of electrocyte abnormalities improved in our two cases by reducing the dose of NM or terminating the administration, and careful attention should be paid to NM-induced hyponatremia and hyperkalemia.

Although further research is needed to ensure the optimal dose and duration of NM administration for efficacy and safety as an anticoagulant during temporary MCS therapy, NM may have a potential benefit as an anticoagulant alternative to heparin or concomitant with low-dose heparin even in patients receiving Impella support.

## Data Availability

Data are available upon reasonable request by the corresponding author.
